# ITRPCA: a new model for computational drug repositioning based on improved tensor robust principal component analysis

**DOI:** 10.3389/fgene.2023.1271311

**Published:** 2023-09-18

**Authors:** Mengyun Yang, Bin Yang, Guihua Duan, Jianxin Wang

**Affiliations:** ^1^ School of Mechanical and Energy Engineering, Shaoyang University, Shaoyang, China; ^2^ School of Computer Science, Hunan First Normal University, Changsha, China; ^3^ School of Computer Science and Engineering, Central South University, Changsha, China

**Keywords:** drug repositioning, tensor robust principal component analysis, weighted k-nearest neighbor, low-rank tensor, drug–disease associations

## Abstract

**Background:** Drug repositioning is considered a promising drug development strategy with the goal of discovering new uses for existing drugs. Compared with the experimental screening for drug discovery, computational drug repositioning offers lower cost and higher efficiency and, hence, has become a hot issue in bioinformatics. However, there are sparse samples, multi-source information, and even some noises, which makes it difficult to accurately identify potential drug-associated indications.

**Methods:** In this article, we propose a new scheme with improved tensor robust principal component analysis (ITRPCA) in multi-source data to predict promising drug–disease associations. First, we use a weighted *k*-nearest neighbor (WKNN) approach to increase the overall density of the drug–disease association matrix that will assist in prediction. Second, a drug tensor with five frontal slices and a disease tensor with two frontal slices are constructed using multi-similarity matrices and an updated association matrix. The two target tensors naturally integrate multiple sources of data from the drug-side aspect and the disease-side aspect, respectively. Third, ITRPCA is employed to isolate the low-rank tensor and noise information in the tensor. In this step, an additional range constraint is incorporated to ensure that all the predicted entry values of a low-rank tensor are within the specific interval. Finally, we focus on identifying promising drug indications by analyzing drug–disease association pairs derived from the low-rank drug and low-rank disease tensors.

**Results:** We evaluate the effectiveness of the ITRPCA method by comparing it with five prominent existing drug repositioning methods. This evaluation is carried out using 10-fold cross-validation and independent testing experiments. Our numerical results show that ITRPCA not only yields higher prediction accuracy but also exhibits remarkable computational efficiency. Furthermore, case studies demonstrate the practical effectiveness of our method.

## 1 Introduction

Over the past few decades, while funding for drug development has seen a substantial surge, the number of newly approved drugs for market release has remained limited. Notably, developing a new drug demands an average of 13.5 years and involves an average expenditure of 1.8 billion (Liu et al., 2020). This process is time-consuming and tremendously expensive and involves high risk (Chong and Sullivan, 2007; Dickson and Gagnon, 2009). Since the approved drugs already possess safety records, tolerance, and pharmacokinetic data of the human body in clinical trials, discovering new clinical indications for commercialized drugs is an important strategy to improve the efficiency of drug development (Ashburn and Thor, 2004). In fact, there have been a few successful repurposed drugs, such as sildenafil, thalidomide, and retinoic acid, which have been widely used in application (Luo et al., 2021).

Using computational methods to discover new uses for established drugs is a crucial aspect of drug repositioning, which is based on the assumption that drugs with similar properties tend to treat similar diseases. With the rapid development of high-throughput technology and continuously generating multi-omics data, there is an increasing focus on crafting computational methods for elevated precision (Wang et al., 2021). These approaches can be classified into four distinct groups: encompassing network-based methods, machine learning-based methods, matrix-based methods, and deep learning-based methods.

Network-based approaches infer the scores of drug–disease pairs by constructing drug and disease heterogeneous biological networks and extracting topological information. The fundamental assumption is guilt by association, whereby if a certain drug can interact with most of the target’s neighbors, it is probable that the target will also be able to interact with the same drug and *vice versa*. Based on the guilt-by-association principle, Wang et al. (2013) used *a priori* information about drugs and targets to establish a heterogeneous graph. A heterogeneous graph-based inference (HGBI) model was used to predict new drug–target interactions. Luo et al. (2016) enhanced the quality of the similarity between drugs and diseases by exploiting the existing drug–disease associations. Building on the combined similarity measures, a new bi-random walk algorithm called MBiRW was developed to infer potential associations between drugs and diseases. Qin et al. (2022) proposed a network-based inference model for new emerging diseases, which used genes as a bridge in a tripartite drug–gene–disease network to infer latent drug–disease associations. Additionally, to account for the structures of networks and the biological aspects related to drugs and indications, Zhao et al. (2022) presented a novel graph representation model based on heterogeneous networks, namely, HINGRL. It integrated the biological networks of drugs and diseases to learn the features from both topological and biological perspectives.

Machine learning-based approaches use supervised learning algorithms to identify potential indications for drugs based on input features and known associations (Vamathevan et al., 2019), including logistic regression (Yang et al., 2021), random forests Zhao et al*.* (2022), and support vector machines (Lavecchia, 2015). Jiang and Huang (2022) proposed a graph representation model based on random forest for drug repositioning. The method identified drug–disease associations by feeding combined features from the molecular association network into a random forest algorithm. Gao et al. (2022) presented a model for predicting associations between drugs and diseases, employing similarity kernel fusion (SKF) to merge diverse similarity kernels for drugs and diseases. This fusion resulted in two integrated similarity kernels, and the scores of association pairs were calculated using the Laplacian regularized least square (LapRLS) algorithm.

Matrix-based methods use the low-rank matrix representation of the drug–disease association space to identify novel associations based on the similarity of their profiles. Yang et al. (2019) developed a bounded nuclear norm regularization (BNNR) method to obtain the low-rank matrix of the drug–disease association. This method efficiently handles noise originating from the drug and disease similarity. Yang et al. (2021) proposed a multi-similarity bilinear matrix factorization (MSBMF) method that dynamically integrated multiple similarities of drug and disease into drug–disease association training. It limited the predicted values of the drug–disease association to non-negative. Huang et al. (2020) proposed a multi-task learning method that used ensemble matrix factorization to predict both treatment associations and non-treatment associations between drug and disease. The proposed method can capture complementary features associated with these two tasks. Yan et al. (2022) proposed a multi-view learning with matrix completion method (MLMC), which is capable of effectively utilizing multi-source similarity matrices. The Laplacian graph regularization was pulled into MLMC to acquire an all-encompassing feature representation derived from the multi-similarity information of drugs and diseases.

Deep learning-based methods typically use neural network models to learn the feature representation of drugs and diseases and use these features to predict new association pairs. Xuan et al. (2019) introduced a bidirectional deep learning model based on the convolutional neural network (CNN) and bi-directional long- and short-term memory (BiLSTM). This framework incorporates both similarities and associations between drugs and diseases in addition to pathways that connect specific drug–disease pairs. This approach effectively integrates raw and topological data between nodes. Combining similarity network fusion (SNF) and neural network (NN) deep learning models, Jarada et al. (2021) proposed a method known as SNF-NN, which was designed to forecast novel drug–disease associations. Yu et al. (2021) proposed a layer attention graph convolutional network model to detect the potential uses of drugs. The model performs graph convolutional processing on a heterogeneous network constructed from drug and disease information, thereby achieving association prediction.

**FIGURE 1:**
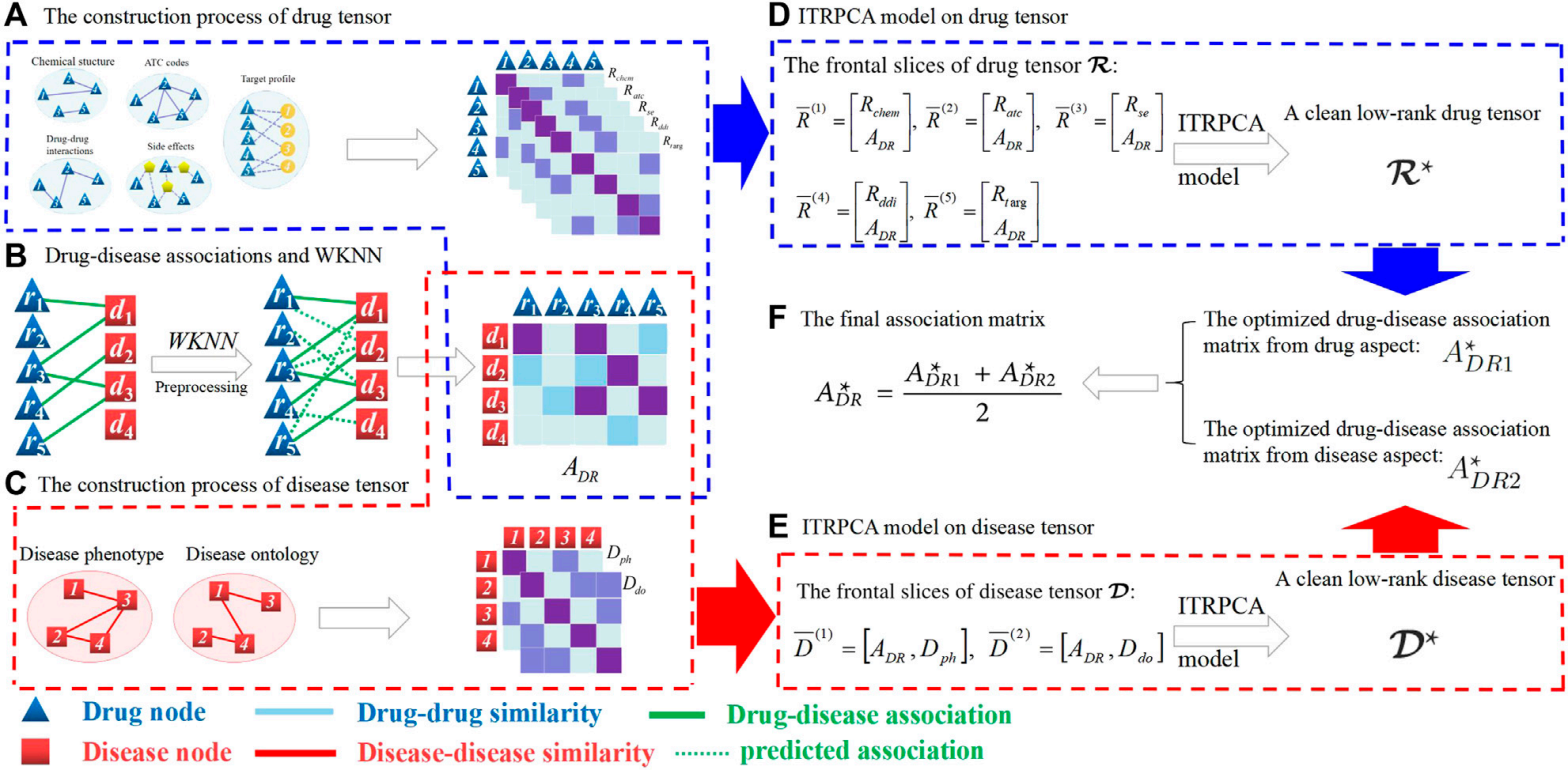
Overall workflow of ITRPCA. **(A)** Construction process of a drug tensor. **(B)** Drug–disease associations and WKNN preprocessing. **(C)** Construction process of a disease tensor. **(D)** ITRPCA model on a drug tensor. **(E)** ITRPCA model on a disease tensor. **(F)** Final association matrix.

To mine latent association features in multiple similarities and association data, we present an improved tensor robust principal component analysis (ITRPCA) method. First, we integrate the prior information of drug and disease to compute five indicators for drug similarity and two indicators for disease similarity. Considering that validated drug–disease associations are extremely sparse, a weighted *k*-nearest neighbor (WKNN) preprocessing step is employed to enrich the association matrix that aids in prediction. Then, we construct a drug tensor and a disease tensor using multi-similarity matrices and an updated association matrix. Finally, we apply ITRPCA to isolate the low-rank tensor and noise information in these two tensors, respectively. We focus on the drug–disease association pairs in the clean low-rank tensor to infer promising indications for drugs. Figure 1 illustrates the comprehensive workflow of the ITRPCA method. Our method’s key contributions are as follows:

• ITRPCA presents a comprehensive scheme for incorporating diverse drug and disease similarities into prediction training.
• By leveraging the weighted tensor Schatten p-norm, ITRPCA can effectively extract the low-rank association tensor from the updated drug and disease tensors, which efficiently separates noisy data and leads to significantly improved accuracy, as demonstrated in our results.
• The ITRPCA model includes a boundary constraint that ensures all predicted tensor entries fall within the predefined interval.
• We have devised an iterative approach employing the augmented Lagrangian multiplier (ALM) to numerically address the ITRPCA model.

## 2 Materials

To validate the effectiveness of our proposed method, this study involves three crucial datasets: the gold standard dataset (Gottlieb et al., 2011), Cdataset (Luo et al., 2016), and CTD (Davis et al., 2019). Table 1 summarizes the details of these three data, such as the number of drugs and diseases, the number of known association pairs, and the intended purposes in this study. These drugs and diseases are obtained from DrugBank (Wishart et al., 2006) and the Online Mendelian Inheritance in Man (OMIM) database (Ada Hamosh, 2005), respectively. The corresponding drug–disease association matrix denoted as *A* is represented by a binary matrix, where the proven drug–disease associations are denoted by 1 s, while unproven associations are denoted by 0 s.

**TABLE 1:** Number of drugs, diseases, and known association pairs in each dataset along with their respective purposes for dataset utilization.

Dataset	# of drugs	# of diseases	# of association pairs	Purpose
Gold standard dataset	593	313	1,933	10-fold cross-validation
Cdataset	663	409	2,352	Independent testing
CTD (February 2020)	1,613	969	15,339	Independent testing

Here, we calculate a total of five similarity matrices for drugs: chemical structure similarity *R*_*chem*_, anatomical therapeutic chemical (ATC) code similarity *R*_*atc*_, side-effect similarity *R*_*se*_, drug–drug interactions similarity *R*_*ddi*_, and target profile similarity *R*_*targ*_. Based on the drug’s canonical SMILES (Weininger, 1988) file, we use the Chemical Development Kit (CDK) (Steinbeck et al., 2003) tool to compute the hashed fingerprints for all drugs and then obtained *R*_*chem*_. ATC codes for all relevant drugs were extracted from DrugBank. We use the semantic similarity algorithm (Resnik et al., 1995) to calculate the similarity scores between ATC terms and then obtained *R*_*atc*_. The rest of the similarities are calculated using the Jaccard similarity coefficient (Jaccard, 1908), which can be expressed as follows:

$$R_{se/ddi/targ}\left(i,j\right)=\frac{\left|S_{i}\cap S_{j}\right|}{\left|S_{i}\cup S_{j}\right|},$$ (1)

where *S*_*i*_ implies the side-effect profiles of drug *i* in *R*_*se*_, drug–drug interaction profiles of drug *i* in *R*_*ddi*_, and drug–target interaction profiles of drug *i* in *R*_*targ*_.

For diseases, a total of two similarity measures are calculated: disease phenotypic similarity *D*_*ph*_ and disease ontology similar *D*_*do*_. *D*_*ph*_ is obtained from MimMiner (Van Driel et al., 2006), which calculates the frequency of MeSH (medical subject heading (MeSH) vocabulary terms co-occurring in the medical descriptions of two diseases retrieved from the OMIM database. According to the structure of the disease ontology academic language, *D*_*do*_ is computed by using the Gene Ontology-based algorithm (Wang et al., 2007).

In summary, we have collected a total of one drug–disease association matrix *A*, five drug similarity matrices (i.e., *R*_*chem*_, *R*_*atc*_, *R*_*se*_, *R*_*ddi*_, and *R*_*targ*_), and two disease similarity matrices (i.e., *D*_*ph*_ and *D*_*do*_) for computational drug repositioning.

## 3 Methods

In this section, we introduce our method for identifying potential uses for established drugs. The structure is as follows: first, we depict the robust principal component analysis (RPCA) and tensor robust principal component analysis (TRPCA). Then, we propose the model of ITRPCA according to the requirements of drug repositioning. At last, the ALM method is demonstrated to solve the ITRPCA model in detail.

For the ease of reference, bold calligraphy letters represent third-order tensors, e.g., $X\in R^{n_{1}\times n_{2}\times n_{3}}$ , capital letters denote the matrices, e.g., *X*, bold lower-case letters indicate the vectors, e.g., ***x***, and lower-case $X_{ijk}$ denote the elements of X .

### 3.1 Robust principal component analysis

RPCA stands as a prominent technique in low-rank representation, which can separate the noise matrix from the original data matrix and learn the clean low-rank matrix. It has found successful applications in computer vision and machine learning, such as video surveillance (Wright et al., 2009), facial modeling (Peng et al., 2012), and subspace clustering (Liu et al., 2010). RPCA is targeted at a matrix, which can decompose the target matrix into a low-rank matrix and a sparse matrix for achieving noise reduction. Generally, the mathematical formula of RPCA can be expressed as

$$\underset{X,E}{\min}\|X{\|}_{*}+\lambda \|E{\|}_{1}\;\text{ s.t. }M=X+E,$$ (2)

where *M* denotes the original matrix, *X* is the low-rank matrix, and *E* is the sparse noise matrix. ‖*X*‖_*_ = *∑*_*r*_ *σ*_*r*_(*X*) represents the nuclear norm of matrix *X*, where *σ*_*r*_(*X*) is the *r*th singular value of *X*. $\|E{\|}_{1}=\sum_{ij}\left|e_{ij}\right|$ denotes the *L*_1_-norm of *E*, and *e*_*ij*_ is the (*i*, *j*) element of *E*.

### 3.2 Tensor robust principal component analysis

TRPCA (Lu et al., 2020) is a continuation of RPCA. The primary motivation behind developing TRPCA is to handle multi-dimensional datasets, which are prevalent in various domains, including computer vision (Wang et al., 2014), object recognition (Zhang and Peng, 2019), and medical imaging (Pham et al., 2021). TRPCA aims to decompose the multi-dimensional data into a low-rank tensor, which captures the essential features of the data, and a sparse tensor, which contains the outliers and noise. The low-rank tensor can be interpreted as the underlying structure of the data, while the sparse tensor represents the deviations from this structure.

Similar to the nuclear norm of the matrix, the tensor nuclear norm (Kilmer and Martin, 2011) is defined as

$$\|X{\|}_{*}=\overset{n_{3}}{\underset{i=1}{\sum }}{\left\|{\bar{X}}^{\left(i\right)}\right\|}_{*}=\overset{n_{3}}{\underset{i=1}{\sum }}\overset{l}{\underset{j=1}{\sum }}\sigma_{j}\left({\bar{X}}^{\left(i\right)}\right),$$ (3)

where $X\in R^{n_{1}\times n_{2}\times n_{3}}$ and $l=\min\left(n_{1},n_{2}\right)$ . ${\bar{X}}^{\left(i\right)}$ is denoted as the *i*th frontal slice of X , and $\bar{X}$ is denoted as the discrete fast Fourier transform (FFT) of X along the third dimension, i.e., $\bar{X}=ifft\left(X,\left[\right],3\right)$ . Thus, $X=ifft\left(\bar{X},\left[\right],3\right)$ . The TRPCA model is formulated as follows:

$$\begin{array}{c} \underset{E,X}{\min}\lambda \|E{\|}_{1}+\|X{\|}_{*}\;\text{ s.t. }M=X+E, \end{array}$$ (4)

where M is the original tensor data, X measures the low-rank tensor, and E denotes the sparse noise tensor. According to Eq. 3, model (4) is equally regularized for all singular values of the tensor data and shrunk with the same parameters when minimizing the tensor nuclear norm.

### 3.3 ITRPCA for drug repositioning

**Weighted** *k*-**nearest neighbor preprocessing**. $\left\{d_{1},d_{2},\ldots ,d_{n}\right\}$ and $\left\{r_{1},r_{2},\ldots ,r_{m}\right\}$ represent the collection of *n* disease nodes and *m* drug nodes, respectively. $A\in R^{n\times m}$ represents the original drug–disease association matrix, where *A*_*ij*_ = 1 if disease *d*_*i*_ is recognized to have a known connection with drug *r*_*j*_; otherwise, *A*_*ij*_ = 0. The *i*th row vector of matrix *A*, i.e., $A_{d}\left(d_{i}\right)=\left(A_{i1},A_{i2},\ldots ,A_{im}\right)$ , represents the association profile of disease *d*_*i*_. The *j*th column vector of matrix *A*, i.e., $A_{r}\left(r_{j}\right)=\left(A_{1j},A_{2j},\ldots ,A_{nj}\right)$ , represents the association profile of drug *r*_*j*_. In fact, if novel drug nodes or disease nodes are considered, the values of their corresponding columns or rows in the adjacency matrix are zero. This case will lead to unsatisfactory performance in prediction (Xiao et al., 2018). We utilize the WKNN algorithm to populate the drug–disease association matrix. This is achieved by considering the similarities of drugs and diseases.

For each drug *r*_*q*_, the similarities of the other *k*-nearest known drugs (where at least one validated association exists) are combined to update the drug’s association profile:

$$\begin{array}{c} A_{r}\left(r_{q}\right)=\frac{1}{Q_{r}}\overset{K}{\underset{j=1}{\sum }}\alpha^{j-1}R\left(r_{j},r_{q}\right)A_{r}\left(r_{j}\right), \end{array}$$ (5)

where the drugs *r*_1_ to *r*_*k*_ are arranged in descending order according to their similarity with *r*_*q*_. *α* ∈ [0, 1] is a decay term, and *R* denotes the mean matrix of five drug similarity matrices. This means that when the similarity between *r*_*j*_ and *r*_*q*_ is strong, a higher weight will be assigned; conversely, a lower weight will be assigned. Furthermore, $Q_{r}=\sum_{1\leq j\leq k}R\left(r_{j},r_{q}\right)$ is the normalization term.

In the same way, the updated association profile for each disease *d*_*p*_ is obtained as follows:

$$\begin{array}{c} A_{d}\left(d_{p}\right)=\frac{1}{Q_{d}}\overset{K}{\underset{i=1}{\sum }}\alpha^{i-1}D\left(d_{i},d_{p}\right)A_{d}\left(d_{i}\right), \end{array}$$ (6)

where *d*_1_ to *d*_*k*_ are the diseases sorted in descending order based on their similarity to *d*_*p*_. *α* ∈ [0, 1] is a decay term, and *D* denotes the mean matrix of two disease similarity matrices. *Q*_*d*_ is a normalization term, and $Q_{d}=\;\sum_{1\leq i\leq k}D\left(d_{i},d_{p}\right)$ . Finishing these profile operations, we obtain the aforementioned two matrices *A*_*r*_ and *A*_*d*_ from drug and disease spaces, respectively. Then, the new drug–disease association matrix *A*_*DR*_ is calculated as follows:

$$\begin{array}{c} A_{DR}=\max\left(A,\frac{A_{r}+A_{d}}{2}\right). \end{array}$$ (7)

After the processing of WKNN, the density of the updated association matrix *A*_*DR*_ is greatly improved, and it no longer contains all zero rows and all zero columns. However, some noise information is inevitably added into the association matrix. Subsequently, we will propose our new method for noise separation.

Algorithm 1 summarizes the preprocessing step for updating the drug–disease association matrix using WKNN.

Algorithm 1 **:** WKNN preprocessing step for updating the association matrix.

• **Input:** The original drug–disease association matrix $A\in R^{n\times m}$ , the five drug similarity matrices: *R*_*chem*_, *R*_*atc*_, *R*_*se*_, *R*_*ddi*_, *R*_*targ*_; the two disease similarity matrices: *D*_*ph*_, *D*_*do*_, decay term *α*, neighborhood sizes *k*.
• **Output:** Optimized association matrix *A*_*DR*_.
1. *R* = (*R*_*chem*_ + *R*_*atc*_ + *R*_*se*_ + *R*_*ddi*_ + *R*_*targ*_)/5, *D* = (*D*_*ph*_ + *D*_*do*_)/2
2. for each drug *r*_*q*_ do
3. $V=KNN\left(r_{q},k,R\right)$ ; $//KNN\left(r_{q},k,R\right)$ is the function to obtain the *k* known nearest neighbors of *r*_*q*_ in matrix *R* in descending order.
4. $Q_{r}=\sum_{j=1}^{k}R\left(r_{j},r_{q}\right)$ ;
5. $A_{r}\left(r_{q}\right)=\sum_{j=1}^{k}\alpha^{j-1}R\left(r_{j},r_{q}\right)A\left(r_{j}\right)/Q_{r};//r_{j}\in V$

6. end for
7. for each disease *d*_p_ do
8. $U=KNN\left(d_{p},k,D\right)$ ;
9. $Q_{d}=\sum_{i=1}^{k}D\left(d_{i},d_{p}\right)$ ;
10. $A_{d}\left(d_{p}\right)=\sum_{i=1}^{k}\alpha^{i-1}D\left(d_{i},d_{p}\right)A\left(d_{i}\right)/Q_{d};//d_{i}\in U$

11. end for
12. $A_{DR}=\max\left(A,\frac{A_{r}+A_{d}}{2}\right)$ ;
13. return *A*_*DR*_.

**Drug tensor and disease tensor.** We construct a third-order drug tensor with five frontal slices denoted as $R\in R^{\left(m+n\right)\times m\times 5}$ . This tensor comprised five drug similarity matrices and an updated association matrix. Specifically, the first frontal slice of the drug tensor is a concatenation of *R*_*chem*_ and *A*_*DR*_, which can be described as follows:

$$\begin{array}{c} {\bar{R}}^{\left(1\right)}=\left[\begin{array}{c} R_{\mathrm{chem}}\\ A_{DR} \end{array}\right], \end{array}$$ (8)

where ${\bar{R}}^{\left(1\right)}\in R^{\left(m+n\right)\times m}$ . In the same way, the remaining four frontal slices of the drug tensor can be constructed with other similarity matrices and *A*_*DR*_, which is presented as

$$\begin{array}{c} {\bar{R}}^{\left(2\right)}=\left[\begin{array}{c} R_{\mathrm{atc}}\\ A_{DR} \end{array}\right],{\bar{R}}^{\left(3\right)}=\left[\begin{array}{c} R_{se}\\ A_{DR} \end{array}\right],\\ {\bar{R}}^{\left(4\right)}=\left[\begin{array}{c} R_{\mathrm{ddi}}\\ A_{DR} \end{array}\right],{\bar{R}}^{\left(5\right)}=\left[\begin{array}{c} R_{\mathrm{targ}}\\ A_{DR} \end{array}\right]. \end{array}$$ (9)

A third-order disease tensor with two frontal slices, namely, $D\in R^{n\times \left(m+n\right)\times 2}$ , is constructed using two disease similarity matrices and an updated association matrix. The disease tensor D is stacked by two slices. Each of its slices can be denoted as

$$\begin{array}{c} {\bar{D}}^{\left(1\right)}=\left[\begin{array}{cc} A_{DR} & D_{ph} \end{array}\right],\\ {\bar{D}}^{\left(2\right)}=\left[\begin{array}{cc} A_{DR} & D_{do} \end{array}\right], \end{array}$$ (10)

where ${\bar{D}}^{\left(1\right)},{\bar{D}}^{\left(2\right)}\in R^{n\times \left(m+n\right)}$ .

**ITRPCA model.** In the two tensors, R and D , some noise is involved in both the similarity data and the inferred association data by WKNN. TRPCA can be employed to separate noise tensors from low-rank tensors. In order to fully exploit the significant information embedded within drug and disease tensors, it is crucial that we adjust the shrinking of large and small singular values such that the large singular values shrink less and the small singular values shrink more. However, TRPCA fails to effectively utilize this prior knowledge during the minimization of tensor nuclear norm. Therefore, the weighted tensor Schatten p-norm is introduced to treat different singular values separately, which is defined as

$$\begin{array}{c} \begin{array}{cc} \|X{\|}_{\omega ,S_{p}} & ={\left(\overset{n_{3}}{\underset{i=1}{\sum }}{\left\|{\bar{X}}^{\left(i\right)}\right\|}_{\omega ,S_{p}}^{p}\right)}^{\frac{1}{p}}\\ & ={\left(\overset{n_{3}}{\underset{i=1}{\sum }}\overset{h}{\underset{j=1}{\sum }}\omega_{j}*\sigma_{j}{\left({\bar{X}}^{\left(i\right)}\right)}^{p}\right)}^{\frac{1}{p}}, \end{array} \end{array}$$ (11)

where $X\in R^{n_{1}\times n_{2}\times n_{3}},h=\min\left(n_{1},n_{2}\right),\sigma_{j}$ denotes the *j*th singular value, and *ω*_*j*_ denotes the weight value of the *j*th singular value. When p = 1 and $\omega =1,\|X{\|}_{*}$ is a special case of $\|X{\|}_{\omega ,S_{p}}$ . Moreover, it is crucial to note that the entries of the low-rank tensor using TRPCA can be any real value in the range of (−*∞*, + *∞*). However, it is imperative to ensure that the predicted values are contextually relevant, as any values falling outside the interval of [0,1] would be meaningless. To address this concern, a bound constraint should be incorporated to restrict the predicted values of unobserved elements within the interval [0, 1]. Our ITRPCA model is formulated as follows:

$$\begin{array}{cc} \underset{E,X}{\min} & \lambda \|E{\|}_{1}+\|X{\|}_{\omega ,S_{p}}^{p}\;\\ s.t. & M=X+E\\ & 0\leq X\leq 1, \end{array}$$ (12)

where M can be replaced by a drug tensor R and disease tensor D in practice.

Here, we use the drug tensor R instead of M as an example. By optimizing the ITRPCA model, a clean low-rank drug tensor $R^{*}\in R^{\left(m+n\right)\times m\times 5}$ can be obtained. Its potential low-rank representation comes from drug multiple similarity data and association information. Actually, we focus on the part of the association tensor in $R^{*}$ , which is denoted as $A_{DR1}^{*}$ and equal to $R^{*}\left(m+1:n+m,:,:\right)$ . In order to obtain a predicted association matrix for inferring potential drug–disease pairs, we take the average of the tensor $A_{DR1}^{*}$ in the longitudinal direction. This operation can be expressed as $A_{DR1}^{*}=avg\left(A_{DR1}^{*},3\right)$ , where $A_{DR1}^{*}$ is the optimized drug–disease association matrix from the drug’s perspective. In the same manner, we substitute the disease tensor D for M in model (12). A new low-rank tensor $D^{*}$ , the other association tensor $A_{DR2}^{*}$ , and the corresponding association matrix $A_{DR2}^{*}$ can be conducted from the perspective of diseases using ITRPCA. It should be noted that $A_{DR2}^{*}=D^{*}\left(:,1:m,:\right)$ and $A_{DR2}^{*}=avg\left(A_{DR2}^{*},3\right)$ . Finally, the integrated drug–disease association matrix $A_{DR}^{*}$ was obtained by averaging the prediction results of both drugs and diseases.

$$\begin{array}{c} A_{DR}^{*}=\frac{A_{DR1}^{*}+A_{DR2}^{*}}{2}. \end{array}$$ (13)

Algorithm 2 summarizes the process of applying ITRPCA in drug repositioning. Based on the predicted pair scores in $A_{DR}^{*}$ , the potential drug–disease association can be inferred.

Algorithm 2 : ITRPCA algorithm in drug repositioning.

• **Input:** Original drug–disease association matrix $A\in R^{n\times m}$ , the mean of drug multiple similarity $R\in R^{m\times m}$ , the mean of disease multiple similarity $D\in R^{n\times n}$ , neighborhood sizes *k*, p-value of Schatten p-norm.
• **Output:** Predicted association matrix $A_{DR}^{*}$ .
1. *A*_*DR*_ ←WKNN preprocessing $\left(A,R,k\right)$ ;
2. Assign R and D by the Eqs (8 − 10).
3. $R^{*}\leftarrow ITRPCA\left(R,p\right)$ ;
4. $A_{\mathrm{DR}1}^{*}{=R}^{*}\left(m+1:n+m,:,:\right)$ ;
5. $D^{*}\leftarrow ITRPCA\left(D,p\right)$ ;
6. $A_{\mathrm{DR}2}^{*}{=D}^{*}\left(:,1:m,:\right)$ ;
7. $A_{\mathrm{DR}1}^{*}\leftarrow avg\left(A_{\mathrm{DR}1}^{*},3\right),A_{\mathrm{DR}2}^{*}\leftarrow avg\left(A_{\mathrm{DR}2}^{*},3\right)$ ;
8. $A_{DR}^{*}=\frac{A_{DR1}^{*}+A_{DR2}^{*}}{2}$ ;
9. return $A_{DR}^{*}$ .

### 3.4 Solutions for ITRPCA

In this subsection, the ALM method is derived to solve the model (12). Accordingly, the augmented Lagrangian function becomes

$$\begin{array}{c} \begin{array}{cc} \Gamma \left(E,X,L,\mu \right)= & \lambda \|E{\|}_{1}+\left\langle L,M-X-E\right\rangle \\ & +\|X{\|}_{\omega ,S_{p}}^{p}+\frac{\mu }{2}\|M-X-E{\|}_{F}^{2}, \end{array} \end{array}$$ (14)

where L is the Lagrange multiplier and *μ* is the penalty parameter. The primary procedure comprises the subsequent distinct subtasks:

$Compute\;E_{k+1}$ :We fix $X_{k}$ and $L_{k}$ to minimize $\Gamma \left(E,X_{k},L_{k},\mu_{k}\right)$ for $E_{k+1}$ . The model (14) becomes

$$\begin{array}{c} \underset{E}{\mathrm{arg min}}\frac{\lambda }{\mu_{k}}\|E{\|}_{1}+\frac{1}{2}{\left\|E-H_{k}\right\|}_{F}^{2}, \end{array}$$ (15)

where $H_{k}=M+\mu_{k}^{-1}L_{k}-X_{k}$ , and drawing inspiration from the soft-thresholding operator, we have

$$\begin{array}{c} E_{k+1}=T_{\frac{\lambda }{\mu_{k}}}\left(H_{k}\right), \end{array}$$ (16)

where the (*i*, *j*, *k*)th element of $T_{\frac{\lambda }{\mu_{k}}}\left(H_{k}\right)$ is $\mathrm{sign}\left({\left(H_{k}\right)}_{i,j,k}\right)•\;\max\left(\left|{\left(H_{k}\right)}_{i,j,k}\right|-\lambda /\mu_{k},0\right)$ .

$Compute\;X_{k+1}$ : We fix $E_{k+1}$ and $L_{k}$ to minimize $\Gamma \left(E_{k+1},X,L_{k},\mu_{k}\right)$ for $X_{k+1}$ . The model (14) becomes

$$\begin{array}{c} \underset{X}{\mathrm{arg min}}\mu_{k}^{-1}\|X{\|}_{\omega ,S_{p}}^{p}+\frac{1}{2}{\left\|X-Y_{k}\right\|}_{F}^{2}, \end{array}$$ (17)

where $Y_{k}=M+\mu_{k}^{-1}L_{k}-E_{k+1}$ . This is a weighted tensor Schatten p-norm minimization (WTSNM) problem based on t-SVD (Kilmer and Martin, 2011). In order to tackle this concern, the subsequent lemma and theorems can be employed.

**Lemma 1** (Xie et al., 2016). For the optimization problem

$$\begin{array}{c} \min_{\delta \geq 0}f\left(\delta \right)=\frac{1}{2}{\left(\delta -\sigma \right)}^{2}+\omega \delta^{p}, \end{array}$$ (18)

with the given *p* and *ω*, there exists a specific threshold

$$\begin{array}{c} \tau_{p}^{GST}\left(\omega \right)={\left(2\omega \left(1-p\right)\right)}^{\frac{1}{2-p}}+\omega p{\left(2\omega \left(1-p\right)\right)}^{\frac{p-1}{2-p}}, \end{array}$$ (19)

and we have the following conclusions:

1) When $\sigma \leq \tau_{p}^{GST}\left(\omega \right)$ , the optimal solution $T_{p}^{GST}\left(\sigma ,\omega \right)$ of Eq. 18 is 0.
2) When $\sigma >\tau_{p}^{GST}\left(\omega \right)$ , the optimal solution is $T_{p}^{GST}\left(\sigma ,\omega \right)=\;\mathrm{sign}\left(\sigma \right)S_{p}^{GST}\left(\sigma ,\omega \right)$ , and $S_{p}^{GST}\left(\sigma ,\omega \right)$ can be obtain by solving $S_{p}^{GST}\left(\sigma ,\omega \right)-\sigma +\omega p{\left(S_{p}^{GST}\left(\sigma ,\omega \right)\right)}^{p-1}=0$ .

**Theorem 1** (Xie et al., 2016). Let $Y=U_{Y}D_{Y}V_{Y}^{T}$ be the SVD of $Y\in R^{m\times n},\tau >0$ , *l* = min (*m*, *n*), 0 ≤ *ω*_1_ ≤ *ω*_2_ ≤ ⋯ ≤ *ω*_*l*_, then a global optimal solution of the following model:

$$\begin{array}{c} \underset{X}{\mathrm{arg min}}\frac{1}{2}\|X-Y{\|}_{F}^{2}+\tau \|X{\|}_{\omega ,S_{p}}^{p}, \end{array}$$ (20)

is

$$\begin{array}{c} {ϒ}_{\tau *\omega }\left[Y\right]=U_{Y}P_{\tau *\omega }\left(Y\right)V_{Y}^{T}, \end{array}$$ (21)

where $P_{\tau *\omega }\left(Y\right)=\mathrm{diag}\left(\gamma_{1},\gamma_{2},\ldots ,\gamma_{l}\right)$ and $\gamma_{i}=T_{p}^{GST}\left(\sigma_{i}\left(Y\right),\tau *\right.$ $\left.\omega_{i}\right)$ , which can be obtained by Lemma 1. The $\left\{\sigma_{i}\left(Y\right)\right\}$ is organized in a descending order, while $\left\{\omega_{i}\right\}$ is arranged in an ascending order.

**Theorem 2** (Gao et al., 2021). Suppose $A\in R^{n_{1}\times n_{2}\times n_{3}},l=\min\left(n_{1},n_{2}\right),0\leq \omega_{1}\leq \omega_{2}\leq \;\cdots \leq \omega_{l}$ , let $A=U*S*V^{T}$ given the model

$$\begin{array}{c} \underset{X}{\mathrm{arg min}}\frac{1}{2}\|X-A{\|}_{F}^{2}+\tau \|X{\|}_{\omega ,S_{p}}^{p}. \end{array}$$ (22)

Then, a global optimal solution to the model (22) is

$$\begin{array}{c} X^{*}={ϒ}_{\tau *\omega }\left(A\right)=U*ifft\left(P_{\tau *\omega }\left(\bar{A}\right)\right)*V^{T}, \end{array}$$ (23)

where $P_{\tau *\omega }\left(\bar{A}\right)$ is a tensor and $P_{\tau *\omega }\left({\bar{A}}^{\left(i\right)}\right)$ is the *i*th frontal slice of $P_{\tau *\omega }\left(\bar{A}\right)$ . $U=ifft\left(\bar{U},\left[\right],3\right)$ and $V=ifft\left(\bar{V},\left[\right],3\right)$ .

According to Theorem 2, the global optimal solution of model (17) is

$$\begin{array}{c} X^{*}={ϒ}_{\mu_{k}^{-1}*\omega }\left(Y_{k}\right)=U*ifft\left(P_{\mu_{k}^{-1}*\omega }\left(\bar{Y_{k}}\right)\right)*V^{T}. \end{array}$$ (24)

In addition, we limit the entry values of $X_{k+1}$ to the interval [0,1] by using the following projection operator.

$$\begin{array}{c} X_{k+1}=Q_{\left[0,1\right]}\left(X^{*}\right), \end{array}$$ (25)

where $Q_{\left[0,1\right]}$ is defined as

$$\begin{array}{c} {\left(Q_{\left[0,1\right]}\left(X^{*}\right)\right)}_{ijk}=\left\{\begin{array}{cc} 1,\; & X_{ijk}^{*}>1,\\ X_{ijk}^{*},\; & 0\leq X_{ijk}^{*}\leq 1,\\ 0,\; & X_{ijk}^{*}<0. \end{array}\right. \end{array}$$ (26)

$Compute\;L_{k+1}$ : We fix $E_{k+1}$ and $X_{k+1}$ to minimize $\Gamma \left(E_{k+1},X_{k+1},L,\mu \right)$ for $L_{k+1}$ . The model (14) becomes

$$\begin{array}{c} L_{k+1}=L_{k}+\mu_{k}\left(M-X_{k+1}-E_{k+1}\right). \end{array}$$ (27)

**Compute *μ***_*k*+1_: In the ITRPCA model, we employ a scheme that gradually increases the learning rate to facilitate fast convergence (Gao et al., 2021). The penalty parameter becomes

$$\begin{array}{c} \mu_{k+1}=\min\left(\rho \mu_{k},\mu_{\max}\right). \end{array}$$ (28)

Algorithm 3 provides the overall iterative scheme of the ITRPCA model. It can extract significant information from the drug tensor and disease tensor and ensure that the predicted drug–disease association values are within [0,1].

Algorithm 3 Solution for the ITRPCA model.

• **Input:** Tensor data M (using drug tensor R or disease tensor D ), *p*-value of Schatten p-norm.
• **Output:** Low-rank tensor X .
• **Initialize**: $X_{0}=E_{0}=L_{0}=0,\mu_{0}=1e-4,\mu_{\max}=1e10$ , *ρ* = 1.1, regularization coefficient *λ* and weight vector ***ω***.
repeat
1. Update $E_{k+1}$ by Eq. 16.
2. Update $X^{*}$ by Eq. 24.
3. $X_{k+1}\leftarrow Q_{\left[0,1\right]}\left(X^{*}\right)$ .
4. Update $L_{k+1}$ by Eq. 27.
5. Update *μ*_*k*+1_ by Eq. 28.
6. *k* ← *k* + 1.
until convergence

returnX .

## 4 Results and discussion

### 4.1 Evaluation metrics

To evaluate the effectiveness of ITRPCA, we employ 10-fold cross-validation to predict potential indications for existing drugs. In this process, known drug–disease associations within the gold standard dataset are randomly split into 10 distinct sets of comparable sizes. One subset serves as the test data, while the remaining nine subsets serve as the training data. This 10-fold cross-validation is repeated 10 times with varied random splits, and the resultant averages are considered the final results. Following prediction generation, potential diseases associated with the test drug are sorted in descending order according to their prediction scores. We utilize three evaluation metrics to evaluate the overall performance of ITRPCA: the area under the receiver operating characteristic curve (AUC), the area under the precision–recall curve (AUPR), and precision.

### 4.2 Parameter setting

In the ITRPCA algorithm, there are some default parameters and two key hyperparameters that need to be adjusted. These default parameters are determined empirically. Specifically, in the WKNN step, we set a decay term *α* equal to 0.95. In model (12), the regularization coefficient $\lambda =\frac{1}{5\sqrt{n_{1}n_{2}n_{3}}}$ , where *n*_1_, *n*_2_, and *n*_3_ are the size of X . For drug and disease tensors, we design an adaptive scheme to determine the weight vector ***ω*** of model (12). We divide ***ω*** into three parts: the first part ranges from 1 to *u*, the second part ranges from *u* + 1 to *v*, the third part ranges from *v* + 1 to the end, and the specific weights of each part are [1, 2, 4]. The number of *u* and *v* are determined by

$$U=avg\left(u_{1},u_{2},\ldots ,u_{n_{3}}\right),V=avg\left(v_{1},v_{2},\ldots ,v_{n_{3}}\right),$$ (29)

where

$$u_{j}=\underset{x}{\mathrm{arg min}}\left\{\frac{\sum_{i=1}^{x}\sigma_{j,i}}{\sum_{i=1}^{m}\sigma_{j,i}}\geq 0.1\right\},\;j=1,2,\ldots ,n_{3},$$ (30)

$$v_{j}=\underset{x}{\mathrm{arg min}}\left\{\frac{\sum_{i=1}^{x}\sigma_{j,i}}{\sum_{i=1}^{m}\sigma_{j,i}}\geq 0.2\right\},\;j=1,2,\ldots ,n_{3}.$$ (31)

Actually, *σ*_*j*,*i*_ is the *i*th largest singular value of the *j*th frontal slice matrix of X . It is evident that by minimizing the weighted tensor Schatten p-norm, the singular values of the second and third parts can be shrunk more compared to the first part. The reason is that these two parts are assigned weight values greater than 1. In addition, the two key hyperparameters are needed to be adjusted, which are neighborhood sizes *k* and p value of Schatten p-norm. We perform grid search to select the appropriate values according to the sum of AUC and AUPR in cross-validation. *k* is chosen from {10, 20, 30, 40, 50}, and p is picked from {0.6, 0.7, 0.8, 0.9, 1}. The numerical results for determining the parameters *k* and p are reported in Table 2. When *k* = 30 and p = 0.9, the highest rating value appears. Meanwhile, we terminate the ITRPCA algorithm when the following stopping criterions are satisfied or the maximum number of iteration steps is reached.

$$f_{k}\leq tol1,\frac{\left|f_{k+1}-f_{k}\right|}{\max\left\{1,|f_{k}|\right\}}\leq tol2,$$ (32)

where $f_{k}=\frac{{\left\|X_{k+1}-X_{k}\right\|}_{F}}{{\left\|X_{k}\right\|}_{F}}$ and *tol*1 and *tol*2 are the given tolerances, which are set as 10^–3^ and 10^–4^ in the algorithm, respectively.

**TABLE 2:** Sum of AUC and AUPR values using different *k*- and *p*-values in the 10-fold cross-validation.

*p* \ *k*	10	20	30	40	50
0.6	1.294	1.299	1.302	1.308	1.312
0.7	1.318	1.327	1.335	1.342	1.348
0.8	1.352	1.365	1.370	1.377	1.378
0.9	1.379	1.391	**1.395**	1.394	1.394
1	1.359	1.364	1.366	1.366	1.365

### 4.3 Comparison with state-of-the-art drug repositioning methods

We compare ITRPCA with five state-of-the-art methods in computational drug repositioning: HGBI (Wang et al., 2013), MBiRW (Luo et al., 2016), BNNR (Yang et al., 2019), MSBMF (Yang et al., 2021), and MLMC (Yan et al., 2022). To ensure a fair comparison, the parameters used in these compared methods are set to the recommended values by the authors (HGBI: *α* = 0.4; MBiRW: *α* = 0.3 and *l* = *r* = 2; and BNNR: *α* = 1 and *β* = 10) or determined by a grid search (MSBMF: *λ*_1_ and *λ*_2_ are chosen from {0.001, 0.01, 0.1, 1}, and *τ* = 0.7; MLMC: *λ*_*r*_ and *λ*_*d*_ are selected from {0.0001, 0.001, 0.01, 0.1, 1}, and threshold = 0.8).

**FIGURE 2:**
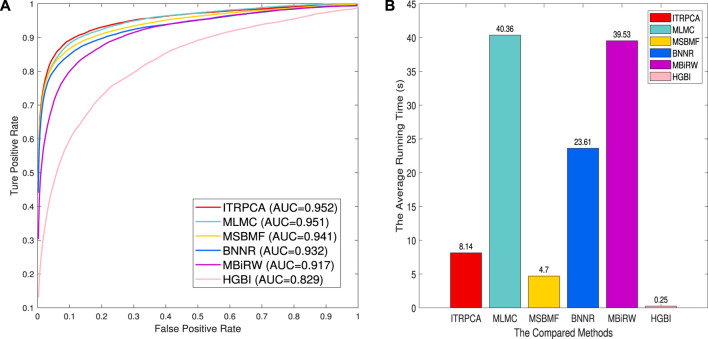
Prediction results of all methods for 10-fold cross-validation on the gold standard dataset. **(A)** Receiver operating characteristic curve of prediction results. **(B)** Average running time for each of the 10 folds.

We assess the performance of all methods in a 10-fold cross-validation for the gold standard dataset. Table 3 shows the AUC, AUPR, and precision values of all compared methods. As shown in Table 3, ITRPCA has the best performance compared to other methods in terms of AUC, AUPR, and precision. Specifically, ITRPCA achieves the best AUPR value of 0.442, which is 67.424%, 4.492%, 4.988%, and 1.376% higher than the corresponding AUPRs of MBiRW, BNNR, MSBMF, and MLMC, respectively. It can be seen that ITRPCA performs slightly better than MLMC. The ROC curves of all methods in the 10-fold cross-validation are shown in Figure 2A.

**TABLE 3:** AUC, AUPR, and precision values of all comparison methods in 10-fold cross-validation for the gold standard dataset.

Metric	ITRPCA	HGBI	MBiRW	BNNR	MSBMF	MLMC
AUC	**0.952**	0.829	0.917	0.932	0.941	$\underline{0.951}$
AUPR	**0.442**	0.102	0.264	0.423	0.421	$\underline{0.436}$
Precision	**0.476**	0.130	0.304	0.463	0.455	$\underline{0.475}$

The most optimal outcomes are indicated in **bold**, while the second-best results are underlined.

Based on the test results from the repeated 10-fold cross-validation, we used the Wilcoxon rank sum tests to evaluate the statistical significance of ITRPCA compared to other methods in terms of AUC, AUPR, and precision. The *p*-values were carefully adjusted using the Bonferroni correction to control for multiple testings. Table 4 shows the *p*-values obtained from the rank sum test and the Bonferroni correction. The results indicate that ITRPCA is significantly better than the other methods, except for MLMC (*p*-value <0.05 ). It suggests that ITRPCA outperforms most of the compared methods in terms of AUC, AUPR, and precision. The significance of the comparison was carefully adjusted using the Bonferroni correction to control for multiple testing.

**TABLE 4:** *p*-values obtained through Wilcoxon rank sum tests and Bonferroni correction, comparing ITRPCA with other methods on AUC, AUPR, and precision.

*p*-value	MLMC	MSBMF	BNNR	MBiRW	HGBI
AUC	1.899	1.125*e*-09	3.019*e*-22	4.932*e*-32	1.281*e*-33
AUPR	1.154	4.741*e*-05	4.741*e*-05	4.741*e*-05	4.741*e*-05
Precision	3.846	4.554*e*-05	1.433*e*-11	1.235*e*-33	1.227*e*-33

In addition, to demonstrate the computational efficiency of the compared methods, we have recorded the average amount of time taken by each fold. The 10-fold cross-validation is executed on a personal laptop, which is powered by an Intel Core i7 processor and comes with 16 GB RAM. Figure 2B shows the average running time for each of the 10 folds across all comparison methods. As shown in Figure 2B, the methods with an average running time of less than 10 seconds are HGBI, MSBMF, and ITRPCA. The average required time for MLMC and MBiRW is relatively long, which is approximately five times that of our method. Therefore, ITRPCA is a promising prediction method that shows both effective predictions and efficient computational performance.

### 4.4 Independent testing

**FIGURE 3:**
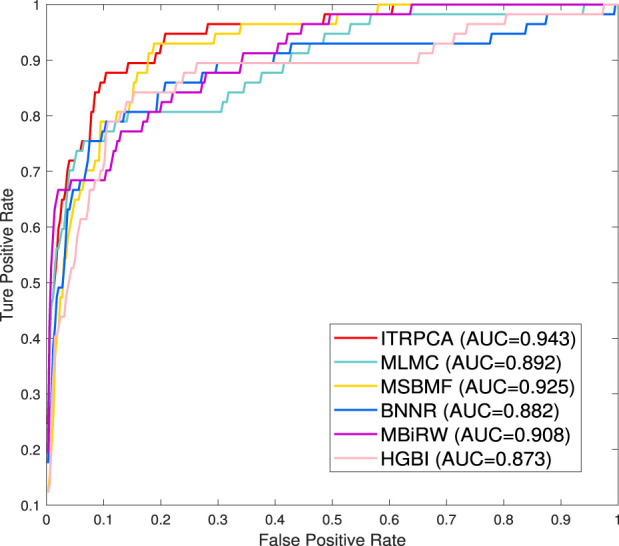
ROC curves for all comparison methods tested independently on the Cdataset.

To further demonstrate the performance of ITRPCA in real applications, we conduct two types of independent testing experiments. The gold standard dataset is used as the training set to train the models, and the set of associated pairs in the Cdataset excluding the training set is used as the testing set to evaluate the performance of the models. To be specific, we have collected a total of 57 drug–disease association pairs in the testing set. Figure 3 shows the ROC curves of all comparative methods in independent testing. As shown in Figure 3, ITRPCA has demonstrated clear superiority over other methods in this independent testing. Specifically, ITRPCA yields an AUC value of 0.943, while HGBI, MBiRW, BNNR, MSBMF, and MLMC yield AUC values of 0.873, 0.908, 0.882, 0.925, and 0.892, respectively. It is worth mentioning that the AUC value of ITRPCA is 5.717% higher than that of MLMC.

**FIGURE 4:**
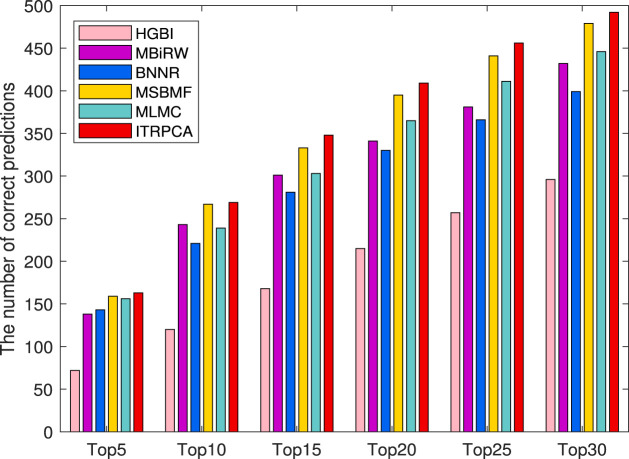
Number of top 5 to top 30 indications correctly predicted for all drugs by all comparison methods in the CTD. The *x*-axis represents the comparison of different methods across six specific top n scenarios. The *y*-axis represents the cumulative sum of confirmed indications among the top n predicted indications for each drug, as determined by the respective methods.

In addition, the other independent testing is conducted using all known associations in the gold standard dataset as training samples and unknown associations as candidate samples. The prediction scores of all candidate pairs are obtained by computational methods and ranked for each specific drug. We focus on how many of the top n candidate indications for each drug could be found and confirmed to have been used in clinical treatment in the CTD (released in February 2020) (Davis et al., 2019). Specifically, among all the drugs and diseases involved in the gold standard dataset, we have identified a total of 938 drug–disease associations that were subsequently validated in the CTD. As shown in Figure 4, the number of correctly predicted associations for 593 drugs is counted for the top 5 to top 30 candidate indications. It is evident that ITRPCA predicts the highest number of correct associations among all the methods for all drugs, followed by MSBMF and MLMC. Specifically, the number of validated associations from the top 5 to top 30 identified by ITRPCA is 163, 269, 348, 409, 456, and 492, respectively. In contrast, the corresponding numbers of identified associations by MLMC are all lower than those by ITRPCA, with a difference of 7, 30, 35, 44, 45, and 46, respectively.

### 4.5 Ablation experiment

To elucidate the individual impact of components within ITRPCA, we designed four ablation experiments: “w/o WKNN,” “only WKNN,” “ITRPCA-drug,” and “ITRPCA-disease.” To be specific, “w/o WKNN” implies the ITRPCA method without WKNN preprocessing for prediction. “only WKNN” represents using only the WKNN algorithm to infer the potential drug–disease associations, without the need for using our tensor RPCA model. “ITRPCA-drug” represents that only the drug tensor in ITRPCA was used to predict drug–disease associations, while “ITRPCA-disease” only uses the disease tensor in ITRPCA. To ensure a rigorous and unbiased comparison, the same prior similarity information and parameters as the ITRPCA model are employed in the aforementioned experiments.

Table 5 shows the AUC, AUPR, and precision results obtained from the 10-fold cross-validation of the comparative methods on the gold standard dataset. As anticipated, ITRPCA performs the best with AUC, AUPR, and precision values. This indicates that combining WKNN and TRPCA has a positive impact on predictive performance. In fact, the “w/o WKNN” model does not exhibit prominent results in predicting latent associations. It illustrates that WKNN preprocessing in ITRPCA can assist in the prediction. For the “only WKNN” model, relevant information was added based on drug and disease similarity. However, this addition also introduced more noise, leading to poor prediction performance. It serves as evidence from the opposite perspective that the effectiveness of TRPCA in noise reduction is significant. Furthermore, based on the prediction results of “ITRPCA-drug” and “ITRPCA-disease,” we find that the simultaneous utilization of tensor information from both drugs and diseases leads to better performance compared to using only one type of tensor information. It implies that the effective enhancement of prediction outcomes can be achieved through the integration of prior knowledge from drugs and diseases.

**TABLE 5:** Compared results of ITRPCA, “w/o WKNN,” “only WKNN,” “ITRPCA-drug,” and “ITRPCA-disease” with 10-fold cross-validation on the gold standard dataset.

Metric	ITRPCA	w/o WKNN	only WKNN	ITRPCA-drug	ITRPCA-disease
AUC	**0.952**	0.929	0.903	0.940	$\underline{0.950}$
AUPR	**0.442**	0.397	0.336	$\underline{0.429}$	0.425
Precision	**0.476**	0.437	0.380	$\underline{0.466}$	0.462

The most optimal outcomes are indicated in **bold**, while the second-best results are underlined.

### 4.6 Case studies

To demonstrate the practical application of ITRPCA, we conducted case studies with the aim of uncovering novel applications for existing drugs. By utilizing all available drug–disease associations and multiple similarities in the gold standard dataset, we applied the ITRPCA method to predict the unexplored relationship between drugs and diseases. Based on the prediction results of ITRPCA, we generated all possible candidate indications for each drug and sorted them according to their obtained scores. In recent years, the development of drugs for tumors and leukemia has received widespread attention. Here, we selected four commonly used anti-tumor drugs ( cisplatin, vincristine, doxorubicin, and methotrexate) and one anti-malignant hematologic drug ( cytarabine) to search for evidence of their candidate indications in the CTD.

Table 6 shows the top 10 candidate indications predicted by the ITRPCA algorithm for the five drugs, with confirmed indications highlighted in bold. It was observed that each drug had 4–6 validated indications among the top 10 predictions. As an example, doxorubicin (DB00997), a broad-spectrum antitumor medication with antibiotic-like properties, was found to be effective in treating various types of cancer, including esophageal cancer, colon cancer, prostate cancer, renal cell carcinoma (nonpapillary), and hepatocellular carcinoma, as shown in Table 6. Additionally, chronic lymphocytic leukemia (susceptibility to, 2) and reticulum cell sarcoma were ranked first and second in the candidate indication list, respectively. However, their validity as indications has not been confirmed yet. These unconfirmed candidate indications hold potential as promising targets for further research.

**TABLE 6:** Top 10 candidate indications for cisplatin, vincristine, doxorubicin, methotrexate, and cytarabine.

Drugs (DrugBank ID)	Top 10 candidate diseases (OMIM ID)
Cisplatin (DB00515)	Rhabdomyosarcoma 2 (268220); **lung cancer (211980);** lymphoblastic leukemia, acute, with lymphomatous features (247640); **diffuse gastric and lobular breast cancer syndrome (137215);** reticulum cell sarcoma (267730); leukemia, chronic lymphocytic, susceptibility to 2 (109543); **Wilms tumor 1 (194070); breast cancer (114480); colorectal cancer (114500);** thyroid cancer, and non-medullary, 2 (188470)
Vincristine (DB00541)	Leukemia, chronic lymphocytic (151400); mycosis fungoides (254400); myelofibrosis (254450); **breast cancer (114480); osteogenic sarcoma (259500); bladder cancer (109800); lung cancer (211980); Kaposi sarcoma, susceptibility to (148000); small cell cancer of the lung (182280);** and diffuse gastric and lobular breast cancer syndrome (137215)
Doxorubicin (DB00997)	Leukemia, chronic lymphocytic, susceptibility to, 2 (109543); reticulum cell sarcoma (267730); **esophageal cancer (133239);** small cell cancer of the lung (182280); testicular germ cell tumor (273300); **colorectal cancer (114500);** Dohle bodies and leukemia (223350); **prostate cancer (176807); renal cell Carcinoma, nonpapillary (144700); and hepatocellular carcinoma (114550)**
Methotrexate (DB00563)	**Lung cancer (211980);** Wilms tumor 1 (194070); **leukemia, chronic lymphocytic (151400); myeloma, multiple (254500); prostate cancer (176807);** renal cell carcinoma, nonpapillary (144700); neuroblastoma, susceptibility to, 1 (256700); thyroid cancer, nonmedullary, 2 (188470); myelofibrosis (254450); and moved to 619182 (175505)
Cytarabine (DB00987)	**Leukemia, chronic lymphocytic (151400);** mycosis fungoides (254400); **myelofibrosis (254450);** rhabdomyosarcoma 2 (268220); **myeloma multiple (254500);** colorectal cancer (114500); small cell cancer of the lung (182280); Kaposi sarcoma, susceptibility to (148000); testicular germ cell tumor (273300); and **breast cancer (114480)**

The predicted indications in **bold** have been confirmed by the CTD.

## 5 Conclusion

In the study, we have proposed a novel computational method called ITRPCA for identifying drug-associated indications. ITRPCA can not only effectively exhibit robustness in isolating the low-rank tensor and noise information but also restrict predicted entry values of the low-rank tensor within a specific interval. The cross-validation and independent testing experiments have shown that ITRPCA is a highly effective prediction method. In particular, when compared to existing drug repositioning methods in independent testing, ITRPCA outperforms them in all measures, indicating a clear advantage. Additionally, case studies have confirmed ITRPCA’s reliability in predicting new indications for known drugs. Therefore, we are confident that ITRPCA will serve as a valuable tool to successfully facilitate practical drug repositioning.

## Data Availability

Publicly available datasets were analyzed in this study. These data can be found at: https://github.com/YangPhD84/ITRPCA.
